# Flat-Silk-Cocoon-Based Wearable Flexible Piezoresistive Sensor and Its Performance

**DOI:** 10.3390/polym16020295

**Published:** 2024-01-22

**Authors:** Zulan Liu, Mengyao Cai, Rui Jia, Xiang Xu, Mengting Xu, Guotao Cheng, Lan Cheng, Fangyin Dai

**Affiliations:** State Key Laboratory of Resource Insects, College of Sericulture, Textile and Biomass Sciences, Southwest University, Chongqing 400715, China; lzlxndx2020@swu.edu.cn (Z.L.); swucmy010703@email.swu.edu.cn (M.C.); jr13057553230@163.com (R.J.); 13606798494@163.com (X.X.); xmengting@163.com (M.X.); cheng_2001@swu.edu.cn (G.C.)

**Keywords:** flat silk cocoon, carbonization, flexible piezoresistive sensor, motion monitoring

## Abstract

Flexible sensors are becoming the focus of research because they are very vital for intelligent products, real-time data monitoring, and recording. The flat silk cocoon (FSC), as a special form of cocoon, has all the advantages of silk, which is an excellent biomass carbon-based material and a good choice for preparing flexible sensors. In this work, a flexible piezoresistive sensor was successfully prepared by encapsulating carbonized flat silk cocoons (CFSCs) using an elastic matrix polydimethylsiloxane (PDMS). The sensing performance of the material is 0.01 kPa^−1^, and the monitoring range can reach 680.57 kPa. It is proved that the sensor can detect human motion and has excellent durability (>800 cycles). In addition, a sensor array for a keyboard based on CFSCs was explored. The sensor has a low production cost and a simple preparation process, and it is sustainable and environmentally friendly. Thus, it may have potential applications in wearable devices and human–computer interactions.

## 1. Introduction

In recent years, with the rapid development of biological materials, related manufacturing technologies, and the accelerated transformation of intelligent manufacturing, the demand for low-cost, large-scale manufacturing and good bio-compatibility flexible intelligent manufacturing is booming. As a key building block of wearable electronic devices, flexible pressure sensors play a vital role in flexible robots, human–computer interaction, and implantable devices [1,2,3,4,5]. A special emphasis is placed on piezoresistive sensors, which have been greatly researched because of their easy designability, flexibility, and simple readout mechanism [6,7,8,9]. The typical preparation strategy is to compose the micro-structuring of the films using different methods for the respective materials [10,11,12,13,14]. Among them, photolithography technology [15,16,17,18], which is the technique of transferring a pattern from the mask plate to the substrate with the help of a photoresist under the action of light, is the most widely used. However, although photolithography techniques yield highly precise and homogeneous patterns, their expensive, complex, and time-consuming processes also prevent their promotion and application in everyday life. Additionally, with the rising concerns over sustainability, researchers are working to develop environmentally friendly and biocompatible materials. Therefore, a piezoresistive sensor with low production cost, good biocompatibility, simple preparation process, and suitable for large-scale manufacturing remains a great research and development interest.

Traditional textiles have the potential to be used as substrates for flexible devices due to their low cost, suitability for large-scale manufacturing, wearability, flexibility, and elasticity [19,20,21,22,23,24,25]. Silk is the softest and finest animal-based fiber in nature, which has a wide range of applications in the medical, industrial, and beauty fields [19,26,27,28]. Unlike ordinary plain silk fabrics, flat silk cocoon (FSC) is a special form of cocoon produced by silkworms. It is made by regulating the cocooning environment of the silkworm so that the silkworm is constrained in the two-dimensional plane, resulting in irregularly spun, interwoven silk in all directions. The FSC has excellent mechanical properties and a unique multilayer structure of cocoons but also breaks the limitations of the size and shape. Meanwhile, choosing flat silk cocoons as substrates for flexible devices does not require additional silk extraction and processing of the fabric. Furthermore, FSC is widely available and less costly, and there are few studies on it. Therefore, it is a good choice as a raw material for sensor preparation. Although FSC does not have good electrical conductivity inherently, it can be transformed into a conductive material through modification while retaining its original integrity. Compared with fiber regeneration and in vivo modification, the carbonization process is simpler and less time-consuming [21,29,30]. In addition, carbon-based materials from natural biomaterials fit well with our research due to their good electrical conductivity, suitability for large-scale manufacturing, and low-cost production capabilities.

Herein, a flexible sensor was obtained by placing the FSC in a tubular furnace filled with inert gas and treating it at high temperature to form conductive carbonized flat silk cocoons (CFSCs), followed by encapsulation with PDMS. In addition, we explored the forms of FSC and carbonization process conditions to obtain flexible piezoresistive sensors with the best performance. The sensor is capable of monitoring human movement and human–computer interaction, which opens up a new avenue for intelligent manufacturing.

## 2. Experimental Section

### 2.1. Materials

A flat silk cocoon was provided by the State Key Laboratory of Resource Insects, Southwest University (Chongqing, China). The polydimethylsiloxane (PDMS) base agent and curing agent was Sylgard 184, Dow Corning (Midland, MI, USA).

### 2.2. Fabrication of CFSCs and the Flexible Piezoresistive Sensor

**Figure 1:**
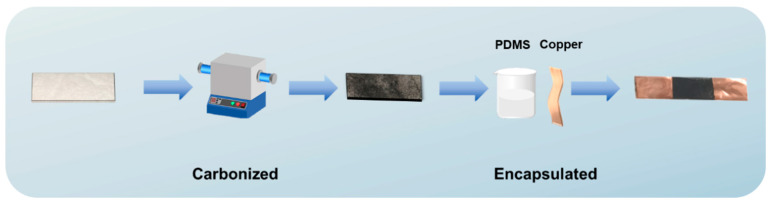
The fabrication scheme of flexible piezoresistive sensor.

The multilayer hot-pressed FSC was cut into 50 × 25 mm strips and heated to 800 °C, 900 °C, and 1000 °C, respectively in a furnace under the flow of inert gas, and then removed after natural cooling. The specific carbonization conditions were first increased at a rate of 5 °C min^−1^ to 150 °C held for 60 min, then raised at the same rate to 350 °C, also held for 60 min, and finally increased at a rate of 2 °C min^−1^ to the target temperature. Thus, the CFSC was obtained. Then, the obtained CFSC conductive films were cut into 30 × 10 mm long strips, and conductive copper foil was pasted at both ends. The width and length of the copper foil electrode connected at both ends of the sensor obtained were 10 mm and 5 mm, respectively, and the distance between the copper foil at both ends was 20 mm. Afterward, the CFSC strips were placed into the prepared mold using PDMS substrate encapsulation (A and B were mixed in a weight ratio of 10:1) and dried in an oven at 60 °C. The fabrication scheme of flexible piezoresistive sensor was shown in Figure 1.

### 2.3. Characterization of CFSCs

The surface morphologies of FSC and CFSCs were characterized using a scanning electron microscope (Phenom microscope, Thermo Fisher Scientific, Eindhoven, The Netherlands, power at 5 kV). The chemical structure of the FSC and CFSCs was characterized by Fourier transform infrared spectroscopy (FTIR, Spectrum 100, PerkinElmer, Waltham, MA, USA). Raman spectra were obtained using a 532 nm diode laser with Renishaw inVia. The surface chemical elements of FSC and CFSCs were analyzed by using a Thermo Scientific Escalab 250 Xi photoelectron spectrograph (Waltham, MA, USA) with a monochromatic Al X-ray source. And the relative atomic concentrations of carbon, nitrogen, and oxygen in the layer of the samples were measured by XPS. 

### 2.4. Measurement of Sensing Performance

The performance of the CFSC piezoresistive sensors was measured with a universal tensile testing machine (MTS SYSTEMS (China) Co., Ltd., Beijing, China, E44.104), while the current signals of the piezoresistive sensor were recorded using an electrochemical workstation (CHI660E instrument, Shanghai, China). The tensile speed of 3 mm s^−1^ was recorded when performing cycling stability.

## 3. Results and Discussion

### 3.1. FSC form Exploration and SEM Characterization

**Figure 2:**
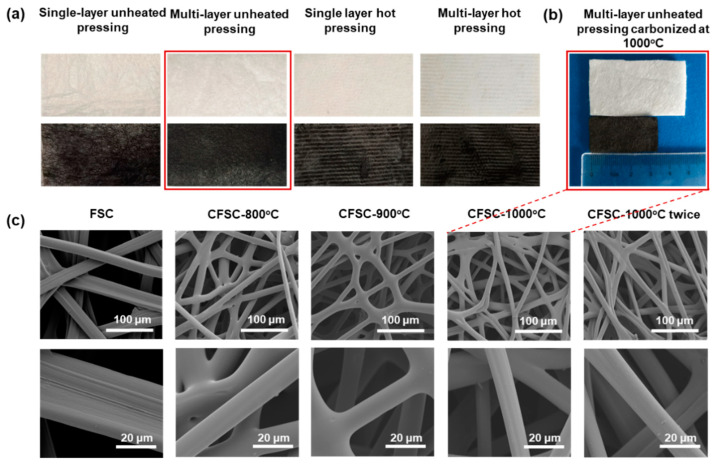
(**a**) Photographs of different forms of FSCs before and after carbonization; (**b**) photograph of a multilayer FSC and CFSC carbonized at 1000 °C; (**c**) SEM images of pristine FSC and CFSCs under different carbonization temperatures.

The photos of different forms of FSC before and after carbonization are shown in Figure 2a. The thicknesses of single-layer cold-pressed, multilayer cold-pressed, single-layer hot-pressed, and multilayer hot-pressed FSCs were 0.117 mm, 0.8 mm, 0.065 mm, and 0.27 mm, respectively. The multilayer FSC had 10 layers, and the hot pressing treatment was conducted at 100 °C and 15 MPa for 5 min. The pristine FSC exhibited a faint yellow color, which transformed into black after carbonization. Both single-layer and multilayer FSCs after hot pressing proved to be brittle and prone to breakage, while the single-layered unpressed FSC also broke with slight pressure due to its thinness. Therefore, the multilayer cold-pressed FSC was selected as the raw material for further experiments due to its multilayered, fluffy, soft three-dimensional structure. As shown in Figure 2b, the area of CFSC obtained by carbonization at 1000 °C had shrunk to about 56% of the original area, while the morphology was still well maintained. In addition, 0.188 g of FSC remained at 0.045 g in mass after carbonization. Typical scanning electron microscopy images of the FSC and CFSCs at different carbonization temperatures are shown in Figure 2c. It can be seen from the figure that after carbonization, the silk fibers were basically not broken, and the network structure of the fabric was not significantly damaged. Compared with the pristine FSC, the fiber diameter became smaller after carbonization, and the fabric structure was more compact.

### 3.2. Chemical Structure Characterization

**Figure 3:**
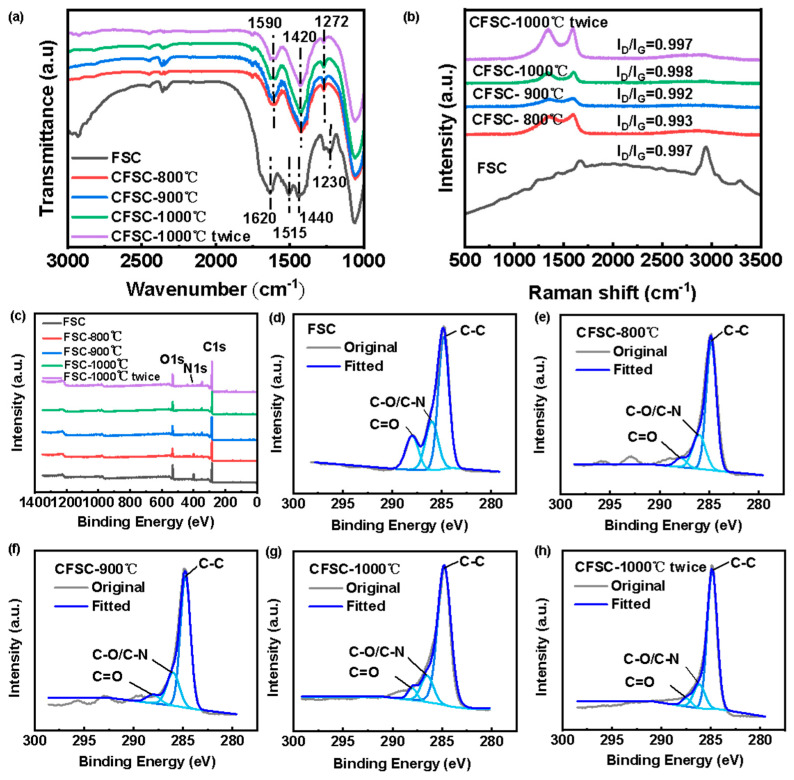
(**a**) The ATR-IR spectra of FSC and CFSCs under different carbonization temperatures; (**b**) Raman spectra of FSC and CFSCs under different carbonization temperatures; (**c**) XPS survey spectra of FSC and CFSCs under different carbonization temperatures; (**d**–**h**) high-resolution C 1s profiles of FSC and CFSCs under different carbonization temperatures: (**d**) FSC; (**e**) CFSC-800 °C; (**f**) CFSC-900 °C; (**g**) CFSC-1000 °C; (**h**) CFSC-1000 °C twice.

To further investigate the chemical structure of CFSC and understand its sensing mechanism, IR, Raman, and XPS spectroscopy techniques were employed, as shown in Figure 3. Typically, silk fibers are composed of millions of silk fibroin molecules. Proteins are biological macromolecules linked by peptide bonds of various amino acids and have unique secondary structures. In the infrared spectrum, these appeared as regions 1700–1600 cm^−1^ (amide I), 1600–1500 cm^−1^ (amide II), and 1350–1200 cm^−1^ (amide III), as shown in Figure 2a. Among them, the characteristic peaks of peptides at 1620 cm^−1^, 1515 cm^−1^, 1230 cm^−1^, and the characteristic peaks of the β-sheet structure in the region of 1622–1637 cm^−1^ appeared only in the IR spectrogram of the original FSC. This indicated that the secondary structure of silk proteins has been changed after carbonization. In addition, CFSC has an absorption peak at 1590 cm^−1^, which is attributed to the C=C or C=N vibrations of aromatic compounds [31]. And the absorption peak at 1420 cm^−1^ is caused by the bending vibration in the C-H plane. The Raman spectra of the samples (Figure 3b) show no significant peaks in the original FSC, while the carbonized samples both show two significant peaks near 1350 cm^−1^ (D band) and 1580 cm^−1^ (G band). The G-peak represents the carbon atomic facets of the hexagonal crystal system and is caused by the in-plane vibration of the sp^2^ carbon atoms, indicating the integrity of the graphite structure. The D peak, on the other hand, is related to the degree of defects and disorder in the graphite structure of carbon materials with crystal defects and irregularities. It can be seen from the figure that the D and G bands of the samples narrow with increasing treatment temperature, indicating the formation of a sp^2^-hybridized carbon structure. The changes in the chemical composition of the silk fabric surface can be analyzed by XPS. Figure 3c shows that after the high-temperature carbonization treatment, the CFSC was still mainly composed of three elements, C 1s (284.8 eV), N 1s (399.8 eV), and O 1s (531.5 eV). Compared with the original FSC, the carbonized C 1s content increased significantly in all CFSCs, while the N 1s and O 1s content decreased. The analysis of the C 1s elements of each sample (Figure 3d–h) showed that the chemical bonds present were mainly C-C, C-N, C-O, and C=O bonds and aromatic structure. C-C is an aliphatic carbon derived from amino acids, demonstrating the presence of graphite-like functional groups. The content of each element and the chemical bonds in the samples are shown in Table 1. This analysis indicates that the percentage of C-C bonds increases while the percentage of C-O, C=O decreases with the increase in carbonation temperature. This proves that during the carbonation process, the adjacent β-sheet crystals in the silk polypeptide are dehydrated, aromatized, or cyclized to form a graphitized structure, which makes CFSC conductive. 

### 3.3. The Sensing Performance

**Figure 4:**
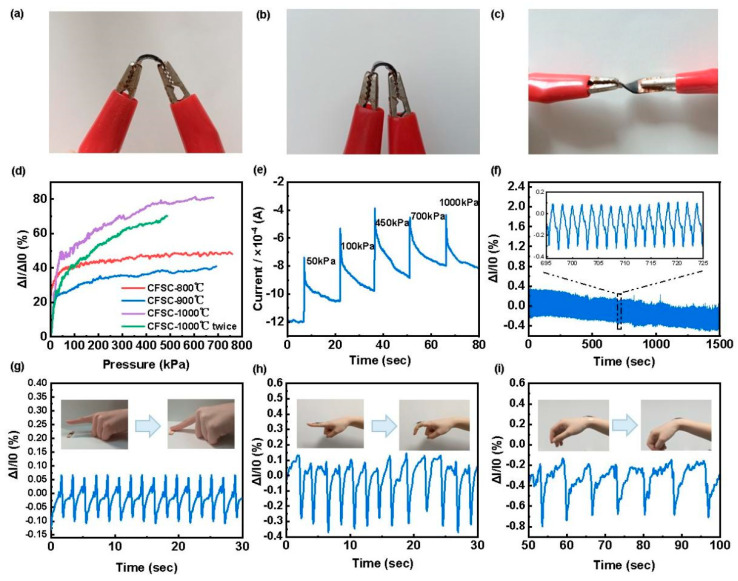
(**a**–**c**) Bending and torsion of flexible piezoresistive sensors; (**d**) the sensitivity of CFSC under different carbonization temperatures; (**e**) the I-t curves of the sensor using CFSC-1000 °C under serial pressures; (**f**) the excellent stability of the sensor after 800 pressure cycling tests under 40 kPa; (**g**–**i**) the signal responses from human movement: (**g**) finger pressing; (**h**) finger bending; (**i**) wrist bending.

In addition, flexible piezoresistive sensors with different carbonization temperatures were obtained by encapsulating CFSCs using PDMS. The prepared piezoresistive sensor has good flexibility and can be bent and twisted, as shown in Figure 4a–c. Sensitivity is an important indicator to determine whether the sensor has a sensing effect. The higher the sensitivity, the better the sensing performance of the sensor. The sensitivities of the CFSCs that were treated under different temperatures are shown in Figure 3d. As can be seen, the sensitivity of samples increases gradually with the increase in processing temperature, and the sensitivity is the highest when the treatment temperature reaches 1000 °C. When the sensor is stimulated by external forces, the CFSC has a tighter structure and more conductive paths, and the sensing effect is more obvious. To verify the reliability of the CFSC-1000 °C sensor, different dynamic pressures were applied to the sensor (Figure 4e). It was clear that the relative current output increases gradually with increasing pressure, indicating that the conductive path of the sensor is gradually increasing. To test the long-term reliability of the sensor, 800 cycles of pressure release tests were performed at a pressure of 40 kPa (Figure 4f). In addition, the sensitivity, detection range, response time, and cycling stability of carbon-based material piezoresistive sensors and CFSC-based flexible piezoresistive sensors are reported in Table 2. In the first stage of CFSC-1000 °C, the sensing performance of the material is 0.01 kPa^−1^. And the monitoring range can reach 680.57 kPa. As shown in Figure 4g–i, the resulting flexible piezoresistive sensor can detect the human motion signals of finger press, bending, and joint flexion. The experiments showed that the sensor can be used in wearable fields such as human motion monitoring.

### 3.4. Human–Computer Interaction

**Figure 5:**
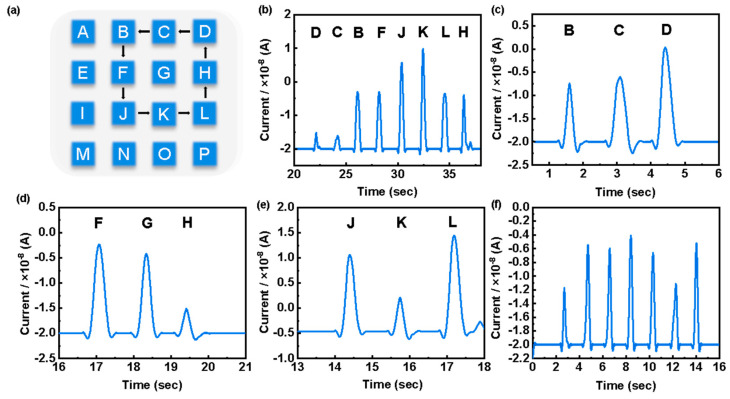
(**a**) A 4 × 4 sensor array and D–C–B–F–J–K–L–H sequential pressure load run diagram; (**b**) signal response of the sensor array during a continuous sequence of pressure loads following the D–C–B–F–J–K–L–H pattern; (**c**–**e**) relative current change intensity obtained by the sensor array at different positions; (**f**) response current when different pressure loads are applied to point D.

To further explore the practicality of this sensor, the prepared CFSCs were cut into 16 square sheets of 10 × 10 mm size and interconnected with copper foil (copper foil width of 3 mm) to produce a 4 × 4 array sensor, as shown in Figure 5a. The relative current response was detected when pressure was applied to different positions of the sensor array in groups of three (Figure 5c–e). In addition, using different forces to press point D, different magnitudes of response currents can be obtained, as shown in Figure 5f. This indicates that the sensor array can determine the location and magnitude of the pressure load based on the response current magnitude. When pressure is applied sequentially to the sensor array along the trajectory shown in Figure 5a, the electrical signals can be output in the order of applied pressure (Figure 5b). Compared with single pressure recognition, array trajectory tracking shows a wider range of potential applications for sensors in the field of human–machine interfaces.

**Figure 6:**
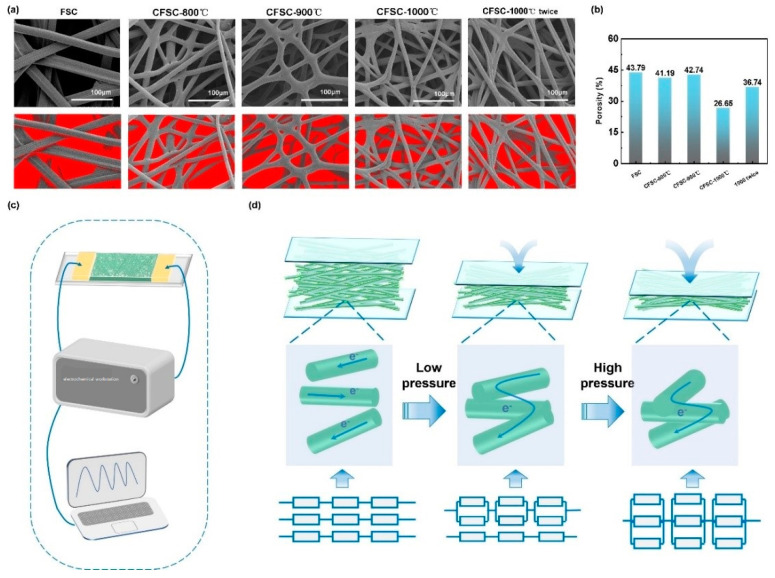
(**a**) Porosity analysis of the FSC and CFSCs under different carbonization temperatures according to SEM images; (**b**) the porosity of FSC and CFSCs under different carbonization temperatures; (**c**) schematic diagram of the electrical signal test system; (**d**) schematic model of the piezoresistive mechanism of CFSC-based multilayer pressure sensors.

To understand the piezoresistive mechanism of the multilayer pressure sensor, we analyzed the porosity of each material and built an intuitive schematic model. By analyzing the porosity of the FSC and CFSCs under different carbonization temperatures (Figure 6a,b), the porosity of the material at 1000 °C is the smallest and the resistivity is small, and thus the sensing performance is good. Therefore, we chose the carbonized material at 1000 °C for testing and analysis. In addition, as shown in Figure 6c, a high-precision electrical signal test system was established to quantitatively evaluate the sensitivity of the sensor and its response to external pressure changes. The response of the sensor under different pressures is displayed as I-t curves by an electrochemical workstation at a bias voltage condition of 0.01 V. A schematic model of the piezoresistive mechanism of the CFSC-based multilayer pressure sensor is shown in Figure 5d. The total resistance (R) of the sensor circuit consists of the volume resistance (R_v_) of the carbonized fibers and the contact resistance between the carbonized fibers (R_c_). Copper foil is a conductive metal, and its resistance can be ignored. The calculation formula is simplified as follows:

R = R_v_ + R_c_

We suppose R_v_ is constant due to carbonized fibers being protected by the PDMS packaging layer, whose volume will not change. When the sensor is under pressure, the carbonized fibers almost withstand all pressures and will deform, which causes the carbonized fibers to come into contact with each other and changes the contact resistance (R_c_). When a smaller pressure is applied to the multilayer pressure sensor, the air layer between the layers of carbonized fibers disappears and the number of contact points inherent in the conductive network formed by the carbonized fibers increases [37]. When the pressure is further increased, the flexible fibers deform elastically and the contact area of each part further increases, resulting in a continuous decrease in resistance. Therefore, by applying different external pressures to CFSCs, the fibers are subjected to different degrees of stress and different resistances, thus showing different current curves. In addition, the use of PDMS to encapsulate the CFSC makes the sensor flexible, and the elastic deformation is reversible and sustainable, which can ensure a long service life.

## 4. Conclusions

In summary, we prepared CFSCs with different temperatures and forms to explore the morphology and carbonization conditions of FSCs. The structural changes were analyzed by IR, Raman, and XPS tests. In addition, to obtain a flexible piezoresistive sensor, the CFSCs at different carbonization temperatures were encapsulated using copper foil and PDMS. The sensing performance of the sensor and its application in monitoring human activities, including finger press, finger flexion, and wrist bending, were demonstrated. Furthermore, a 4 × 4 array sensor was prepared to further demonstrate its application in human–computer interactions. This exploration not only expands the choice of conductive raw materials and breaks the limitation of cocoon size but also provides a new idea for the preparation of intelligent devices. Considering the large-scale, low-cost manufacturing process and the requirement of green sustainability, we believe it will show great potential for applications in wearable electronic devices and human–machine interactions.

## Figures and Tables

**Table 1:** Relative chemical composition and atomic ratio determined by XPS for FSC and CFSCs under different carbonization temperatures.

Samples	Chemical Composition (%)			The Relative Area of Different Chemical Bonds (%)			Atomic Ratio C/O
	C1s	N1s	O1s	C–C	C–O/C–N	C=O	
FSC	68.06	13.88	18.07	62.22	23.37	14.40	3.77
CFSC-800 °C	83.98	6.41	9.61	70.21	24.02	5.77	8.73
CFSC-900 °C	87.58	2.43	9.99	71.03	22.59	6.38	8.76
CFSC-1000 °C	85.85	1.40	12.75	79.37	15.06	5.57	6.73
CFSC-1000 °C twice	88.65	0.94	10.41	79.21	16.02	4.77	8.52

**Table 2:** Sensing performance comparison of CFSC-based flexible piezoresistive sensors and reported piezoresistive sensors.

Reference	Sensitivity (kPa^−1^)	Detection Range (kPa)	Response Time (ms)	Repeatability (Cycles)
[31]	103.5	0.01	——	50,000
	27.5	18		
[12]	6.04	700	——	1000
[32]	100.29	2	——	11,000
	21.22	10		
[33]	0.585	35	4	5800
[14]	13.89	6	64	500
[34]	−2.52	0.16	20	——
	−0.2	1.2		
	−0.01	9		
[35]	33.96	5	——	6000
	1.22	20		
[36]	37.5	2	50	3000
This work	0.01	680.57	——	800

Note: —— means null.

## Data Availability

Data are contained within the article.
